# Developing a More Tailored Approach to Patient and Public Involvement with Children and Families in Pediatric Clinical Research: Lessons Learned

**DOI:** 10.1007/s43441-022-00382-4

**Published:** 2022-02-19

**Authors:** J. Preston, B. Nafria, A. Ohmer, S. Gaillard, P. Dicks, L. West, M. A. Turner

**Affiliations:** 1 Faculty of Health and Life Sciences, Women’s and Children’s Health, Liverpool Health Partners, University of Liverpool, Liverpool, UK; 2 Institut de Recerca Sant Joan de Déu, Spain and Innovation and Research Department, Hospital Sant Joan de Déu Pg, Santa Rosa 39-57, Sant Joan de Déu, 2, Esplugues de Llobregat, 08950 Barcelona, Spain; 3 International Children’s Advisory Network, Atlanta, GA USA; 4 Hospices Civils de Lyon, EPICIME-CIC 1407 de Lyon, Inserm, CHU-Lyon, 69677 Bron, France; 5 NHS-NRS Children, NHS Grampian, Royal Aberdeen Children’s Hospital, Aberdeen, AB25 2ZG Scotland UK; 6 Georgia Institute of Technology, and International Children’s Advisory Network, Atlanta, GA USA; 7 Department of Women’s and Children’s Health, Institute in the Park, University of Liverpool, Alder Hey NHS Children’s Foundation Trust, Liverpool, UK

**Keywords:** Pediatric clinical research, Children, Families, Involvement, Patient-centricity

## Abstract

Listening to, and acting on, the voices of children and families during clinical research and innovation is fundamental to ensuring enhanced pediatric health care, medicines development, and technological advances. While this is often discussed as an important step in ensuring patient-centered care, involving children and families across the life cycle of clinical research is not currently routine. The pediatric research community needs to address how to meaningfully involve children and families if they are to succeed in designing clinical research that suits the needs of pediatric patients and their families. This paper describes how an international community working under the umbrella International Children’s Advisory Network (iCAN) and European Young Person’s Advisory Group Network (eYPAGnet) has involved children and families in the design and delivery of pediatric clinical research. It offers practical solutions through various case studies assessed against seven patient engagement quality criteria within the Patient Engagement Quality Guidance (PEQG) tool, highlighting some of the lessons learnt from involving and engaging with children and families across different stages of clinical research, including pediatric trials for drug development programs.

## Background

Undertaking pediatric clinical research presents unique challenges. Barriers to successful study completion include parental and patient expectations, motivations, and attitudes regarding the benefit and burden of study participation; a higher rate of early patient drop-out; the inherent characteristics of the pediatric population; and the intensity of study procedures and associated demands on patients and families. These barriers all contribute to reduced patient participation or retention, resulting in far fewer therapeutic advances for children and young people [1]. One way to overcome some of these barriers is to involve children and families throughout the research and development process, from identifying unmet needs and patient priorities through to the dissemination of study findings. This involvement also respects the rights of children and young people.

The potential influence and impact that patients and the public (including children and young people) have on the design and conduct of clinical research are increasingly being recognized by researchers, funding bodies, and journal editors. In health and social care research, this is often badged under the umbrella term ‘Patient and Public Involvement’ (PPI), which has been defined as *“research…carried out ‘with’ or ‘by’ members of the public rather than ‘to’, ‘about’ or ‘for’ them”*. [2]

While policy and evidence strongly advocate for the active and meaningful PPI in clinical research, it is more difficult to define how this is embodied in practice, especially where children are concerned. Most of the published literature focuses on PPI work with adults and the reporting of children and family involvement in clinical research being scarce [3–5]. As a result, understanding of how involvement works for children and families, what the key challenges are, and what needs to be in place to make it meaningful for those involved is limited [6].

Those who work with children and families understand that involving them meaningfully requires tailoring practices and methods to suit their needs and requirements [7]. Involving children, in particular, is complex and multi-dimensional, requiring consideration of four key areas:

- Level of participation (degrees of power-sharing between adults and children),
- Focus of decision-making (individual or collective),
- Model of participation (consultation, collaboration, child-led), and
- Clarity of the term ‘children’ which covers a diverse group who are not only different in their personal circumstances (age, sex, ethnicity, culture, disability, social and economic circumstances) but their changing interests and capacities as they grow older [8].

We write this paper as an international community of advocates for children and young people (hereon children) and family involvement in all stages of the research process. The goal of this paper is to share our lessons learned and practical tips to enhance meaningful PPI with children and families. All authors are linked in various capacities to the International Children’s Advisory Network, Inc. (iCAN) http://icanresearch.org/ and the European Young Person’s Advisory Group Network (eYPAGnet) http://eypagnet.eu/ who work alongside pharmaceutical companies, academic researchers, regulatory agencies, ethics committees, and others to make sure children and families are involved in the decision-making processes [9]. The networks are renowned for working in partnership with children through the forum of a Young Person’s Advisory Group (YPAG), which first emerged in 2006 in the UK [10]. Both networks are led by the principle that YPAG activities should transform children from research subjects into research partners. Members of eYPAGnet and iCAN YPAGs normally consist of a mix of children between the ages of 8–18 years old with either experience of having a chronic condition, hospitalization, clinical trial participation, or a general interest in science and research [11]. YPAGs are stable organizations that provide opportunities for members to encounter clinical research and learn about some key features of research that they can influence. The networks also have experience of working with other, ad hoc, groups of children and families (who are not members of YPAGs) from various backgrounds, interests, and experiences of childhood illnesses as and when required [12].

Published principles or quality PPI standards [13] may tell us what we should aspire to. However, they often lack practical details on implementing PPI in practice, especially with children and families [14]. To share our experience with the practicalities of involving children and families throughout the research process, the authors have used a Patient Engagement Quality Guidance (PEQG) tool [15] as a guide to reflect on the lessons learned from PPI with children and families. The PEQG tool was developed by the Patient Focused Medicines Development initiative, a not-for-profit collaborative organization to benefit patients and health stakeholders by encouraging patient-centered healthcare systems [16]. The tool was developed with over 70 experts in 51 organizations as a practical guide to planning, developing, and assessing the quality of patient involvement activities and projects. The tool contains seven quality measures, which include the following: (1) Shared Purpose, (2) Respect and accessibility, (3) Representativeness of stakeholders, (4) Roles and responsibilities, (5) Capacity and capability for engagement, (6) Transparency and communications and documentation, and (7) Continuity and sustainability. A brief description and rationale for each measure can be found in Table 1.

**Table 1:** Patient Engagement (PE) quality criteria summary and description

PE quality criterion*	Brief description and rationale
1. Shared purpose	*Brief description*: Shared purpose refers to the importance of all stakeholders agreeing on the project’s aims and outcomes before starting the project
	*Rationale*: Early involvement is a key factor for the quality of the process and includes the consideration of all perspectives in the early phase of planning
2. Respect and accessibility	*Brief description:* Respect and accessibility refer to (1) respecting each other and respectful interactions within the project to be established among partners, and (2) openness to and inclusion of individuals and communities (to the project) without discrimination
	*Rationale*: A key quality aspect is the importance of securing a supportive culture that reflects that all stakeholders acknowledge the patients’ perspective (at any age) as equally important to that of other professional or authoritative stakeholders. Practical steps must be taken to ensure access for all
3. Representativeness of stakeholders	*Brief description*: Representativeness of stakeholders refers to the mix of people involved, which should reflect the needs of the project and the interests of those who may benefit from project outputs
	*Rationale*: Ensuring optimal representativeness is demanding but essential for any PE activity and involves careful consideration of the selection of patient representatives. For example, appointed patient representatives in committees may often be particularly resourceful relating to their disease and treatment, and it becomes important to ensure perspectives of less resourceful or vocal patients are considered
4. Roles and responsibilities	*Brief description*: Roles and responsibilities refer to the documentation of agreed, and ideally co-created, roles and responsibilities, indicating that all aspects of project needs will be established upfront and revisited regularly
	*Rationale*: Clarity on roles and responsibilities of all partners is essential for the implementation of equitable working practices that ensure PE opinions and expertise are respected and incorporated, where possible, into PE projects. It is understood that not all feedback was given by young people and families can be incorporated into the end product due to legal limitations
5. Capacity and capability for engagement	*Brief description:* Capacity and capability for engagement refer to (1) capacity as having relevant and dedicated resources from all stakeholders and (2) capabilities for all stakeholders to enable meaningful engagement
	*Rationale*: It is essential that everyone has sufficient knowledge and skills to contribute effectively. These skills include the professionals having sufficient PE knowledge and skills, as well as patients having sufficient trials knowledge
6. Transparency in communication and documentation	*Brief description:* Transparency in communication and documentation refers to the establishment of a communications plan and ongoing project documentation that can be shared with stakeholders. Communication among stakeholders must be open, honest, and complete
	*Rationale*: Transparent communication throughout the project, both internally and externally, is essential to ensure the credibility of the process and findings. Publication of protocols and results of all trials are increasingly recognized as essential for the effective and ethical evaluation of clinical products
7. Continuity and sustainability	*Brief description:* Continuity and sustainability refers to the smooth progression of the project and efforts to maintain relationships with stakeholders beyond a single project
	*Rationale*: Involvement of patients throughout the process as much as feasible, including aspects such as evaluation, dissemination, and implementation can be very beneficial for the quality of the process. Additionally, ongoing commitment to PE and the development of long-term relationships will enhance the quality of outputs. Also, the long-standing relationships that iCAN and YPAGs have with their children and young people beyond a specific activity facilitate many aspects of all engagements

Accordingly, the aims of this paper are as follows:

1. Summarize pertinent case studies
2. Identify lessons learnt and common themes from the case studies
3. Review the quality of the case studies in the light of the PEQG tool

## Methods

Eleven PPI activities with children and families were identified by the authors and assessed using the PEQG tool. The activities were purposively chosen to provide a selection of requests received to partner with children and families from regulatory bodies, life science and biotech companies, and academic institutions (hereon referred to as researchers). Six of the authors (4 from eYPAGnet, and 2 from iCAN Inc) responsible for coordinating and facilitating children and family involvement completed 14 case study templates (see Table 2) and shared them with the wider authorship team before agreeing on the final eleven examples. Three case studies were excluded as they focused on engagement activities (e.g., inviting children to conferences or producing a video to tell their story or to promote the importance of involving children in clinical research) as opposed to focusing on the actual involvement activity itself. Attention was paid to selecting cases that could represent a variety of activities across different stages of the research process, working with different populations. Each case study was reviewed by the team to ensure that the guidance linked to the PEQG tool was systematically followed to ensure an open and transparent reporting process that highlighted both positive and negative lessons learned.

**Table 2:** Case study template—assessed against PFMD patient engagement quality guidance

Project/activity title and date of completion
What was the project/activity? brief description
Why was it important to partner with children, young people, and/or families?
How was the project/activity done—what was involved, process, timelines as identified by comparison with PFMD criteria? These will be identified by comparing against a PPI quality framework
PFMD criteria 1: Shared purpose
PFMD criteria 2: Respect and accessibility
PFMD criteria 3: Representativeness of stakeholders (The mix of people you involve should reflect the needs of the project and the interest of those who may benefit from project outputs)
PFMD criteria 4: Roles and responsibilities (Was there clear roles and responsibilities established upfront. Did everybody understand what was required of them? And was this revisited regularly?)
PFMD criteria 5: Capacity and capability for engagement
PFMD criteria 6: Transparency in communication and documentation
PFMD criteria 7: Continuity and sustainability
What were the benefits/challenges for the study/organisation and children, young people, and/or families?
What were the gaps, as identified by comparison with PFMD criteria? (These will be identified by comparing against the PPI quality framework
Learnings and improvements that could be made for future projects

## Results

Of the eleven activities, five activities fell under the category of research priority setting. Three involved children and/or families throughout all stages of the research process and three activities fell under the category of dissemination, communication, and post-approval phases. The types of involvement included virtual panel meetings, Delphi surveys, focus groups, study management membership, consensus meetings, and workshops. Eight of the eleven case studies involved children only: one involved parents only, and two involved both parents and young adults. Table 3 summarizes the eleven activities including who was involved, outcomes, and gaps in practice. A link to a full description of the case studies is included in Table 3.

**Table 3:** Full description of the eleven Case Studies

Case study	Stage of research (research priorities; research design, and planning; research conduct and operations; dissemination, communication, post-approval)	Type of involvement	Who was involved	Outcomes	Things that need improving (gaps in practice)	Link to full Case Study
Funding decision-making for a digital solution for abdominal pain in children and young people	Research priorities	Virtual funding panel consisting of adult professionals including pediatricians, innovations leads, psychologists and 2 young people	2 young people (17 and 21, both female) from an existing Young Person’s Advisory Group	Active representation of youth members on a panel to select projects that received funding for a health application that was intended for use by children	Timing of meetings predetermined by project timetable and panel availability. Professional working week conflicts with YPAG availability requiring them to be absent from school/university	https://bit.ly/3tRyJDH
Identification of unmet medical needs from pediatric patients with a rare condition	Research priorities	Focus group with teenager patients living with an ultrarare disease	7 female patients from 12 to 16 years old	● Description of main characteristics of the patient journey ● Formulation preferences from the patients ● Acceptability of placebo ● Home nurse service ● Medical assessments were potentially included in the study. ● Acceptability of the screening process. ● Assessment of the tool to collect feedback about the palatability of the drug.	● The activity was performed with patients from only one hospital. Diversity was not included in the concept design of this activity. The sponsor didn’t see any concern about it ● Still awaiting feedback from the sponsor about if the outcomes were implemented so they can be shared with the patients.	https://bit.ly/3k5UlZJ
Feasibility study	Research design and planning; research conduct and operations; dissemination and communication.	Virtual meetings were held with parents who had experience of neonatal care or paediatric intensive care, Study Management Group Membership, and consensus meeting attendance.	Nine parents (five PICU parents, two NNU parents and two parents having had experience of both NNUs and PICUs)	● Provided valuable insights into the important issues for parents and carers for a future study ● Revision of the lay summary. ● Co-produced Patient Information Sheets, and interview questions. ● Co-produced a summary of the research findings in a visual abstract format and disseminated widely among the PICU and NNU parent communities.	Meeting face to face was extremely challenging for this group of parents. Study teams need to adapt their approaches to suit the demanding lifestyles of parents if they are to be involved meaningfully.	https://bit.ly/3k7DDZC
Core outcome setting for children with chronic kidney disease	Research priorities	Delphi Survey with chronic kidney disease patients. Two to three rounds of surveys were completed sequentially	72 patients around the world, 66% female age range : 8–14 (24, 12%), 15–18 (25, 12%), 19–21 (16, 9%) including French participants.	● Provide reliable consensus on core outcome sets across a range of chronic kidney disease, with an appropriate and dedicated methodology. ● Experts including children and young people, caregivers, and professionals. ● Establishment of a consensus core outcome set for paediatric nephrology trials, to improve quality of reporting and relevance of trails conducted in children and adolescents with chronic kidney disease.	● Challenging to obtain enough children and young people with CKD disease notably because of late participation of France in the process. ● More information developed by and for children and young people to better target them to participate was required. ● Evaluation of the patient’s experience is needed.	https://bit.ly/3kdKr8s
Core outcome set development	Research priorities	2 workshops were held during the International Children’s Advisory Network (iCAN) Research and Advocacy Summit held in 2018	70 young people (aged 10-18 years with a mix of healthy young people and those with experience of acute and chronic conditions).	● Children and young people provided valuable insights and recommendations to optimise core outcome set development with children and young people. ● Feedback from the workshops identified ways to better engage children and young people as participants in core outcome set studies, which will potentially improve the quality of such studies in the future.	Despite many children and young people taking part in the workshops, most of these came from high-income socio economic backgrounds within countries across Europe and North America. Future workshops or consultations would benefit from including children and young people from rarely included groups, such as black and minority ethnic groups and those from socio-economically disadvantaged backgrounds	https://bit.ly/3tJvwWz
Age-appropriate Patient-Reported Outcomes and Quality of Life Scales	Research priorities	Focus group with teenager patients living with an euro muscular rare condition and their parents	4 teenagers (11-18 years old, all female),and 5parents (4female, 1 male).	● Description of the patient journey. ● Preferences about treatment doses and administration. ● Preferences on QoL and PROMs scales. ● Information and educational materials for patients/families.	● All the patients and parents were from the same hospital. Diversity was not a key element in the design of this activity due to the limitations to recruit patients with his condition. ● Need for further information and materials to support the involvement of patients and parents in the activity.	https://bit.ly/2Xm7EMX
Randomised Controlled Trial investigating the use of a diagnostic technique to predict asthma exacerbations in children	Research design and planning; research conduct and operations; dissemination and communication.	YPAG (Scotcrn) 50%of the group had been recruited from a birth cohort that had studied the prevalence of asthma and wheeze since birth.	Group age range was 14–18 years.	Involved from planning stages and provided advice on logo, description of methodology, recruitment, patient information and lay summary.	No formal evaluation or record of the PPI experience from both CTU and YPAG perspective.	https://bit.ly/3Ce4yth
Developing multimedia interventions with children, young people, and families	Research design and planning; research conduct and operations; dissemination and communication.	YPAG (Generation R Liverpool) and formation of a Patient and Parent Advisory Group (PPAG)	12 young people(8–21 years old, 7female, 3 male); 3young adults between 19 and24 years (two female, one male) with long term health conditions, and three parents (all female) of young people with long term health conditions joined the PPAG	● Input and improvement to the patient-facing study documentation. ● Review of multimedia and written content for the multimedia interventions. ● Contributed to decisions regarding study design and governance. ● Promoted the study via social media. ● Contributed to the writing of presentations and publications arising from the TRECA study.	● Meeting face to face is challenging. ● PPAG members would have liked to meet the study team much earlier on in the process to form relationships. ● Paying young people and parents for their time is complicated. Having a payments policy and sharing this with members at the beginning is important. ● Diversity was an issue, involving younger age children was difficult and needs more consideration when planning PPI activities.	https://bit.ly/3tD0dwH
Reviewing documentation for participants to a clinical trial	Research design and planning; Patient information	YPAG Kids France focus group activity, advice on information documents for a future trial in a rare disease condition. Presentation and explanations about he trial and review of the documents	8 young people12 to 19 years old	● Input on the explanations and documentation of assent/consent given to participants to a future trial ● Changes proposed to improve the understanding of the trial design and the conduct of the study. ● Review of the content and readability	● Impact on authorizations was evaluated, but the impact on understanding, and recruitment/retention was not evaluated and missing. ● Experience of the participants and the Principle Investigator about the activity was not evaluated at the end of the involvement.	https://bit.ly/399e9VE
Good Lay Summary Practice- European Guidance	Dissemination, communication, and post approval phase	Face to face meeting with members of existing YPAGs and individual questionnaire	Albania (N = 11) France (N = 8) Italy (N = 7) Scotland (N = 4) Spain (N = 14)	● Recommendations for the pediatric lay summaries ● Visual format is preferable to text format. ● Comics, animations, and infographics were selected as the best format options for children between 12-18 years old. ● A glossary of terms will be useful to clarify and offer extended information about medical terms. ● Seeking the views of young people about the readability will make information more accessible to this age group.	● All the lay summaries were available in English not in the native languages of the 4 to 5 of the YPAGs involved. This can be a limitation to some young people due to their level of competence in the use of English. ● Further involvement of YPAGs from other European countries was desirable. ● Diversity was not a mandatory element in the design of the activity. ● Patient organisations representing pediatric conditions were involved during the public consultation of the guidance after this activity.	https://bit.ly/3nrs2qp
Lay summary of findings	Dissemination, communication, and post approval phase	iCAN completed the first lay summary video supported by one young person from iCAN KIDS Central Ohio	Pediatric Trials Network (PTN), International Children’s Advisory Network, Inc. (iCAN), One young person, a male, age 14.	● Provide valuable insight to researchers regarding the young person's perception, understanding, and clarity of the written lay summary. ● Offer a new method of dissemination of lay summary materials to young people and their families of the content within the lay summary so that it would be more understandable to others.	● Offer additional time to complete lay summary videos. ● Ensure that the young person is included at the beginning of the written lay summary to ensure the appropriateness of reading level, and content. ● Create more opportunities to develop lay summary videos to further advance new communication styles of lay summary to young people and their families.	https://bit.ly/3tJxk1S
Working with Regulators	Research design and planning; research conduct and operations; dissemination and communication.	Face to Face meeting, including a panel of youth members and US Food and Drug Administration (FDA)regulators	6 children with diagnosed rare conditions (including 5 female, 1 male) in the ages of 8-19 years old.	• Youth Input on the patient experience through sharing insight of their own personal rare disease journey. • Youth input focused on patient endpoints. • Heavy focus on accessibility, new medical treatment and medical device needs for all young people. • Feedback from youth members supported improvements within clinical research trial development, regulatory processes, and patient centred care for improved treatments and new medicine development.	• Experience and feedback of patient involvement was not evaluated. • While parents/guardians were invited to chaperone and attend the meetings, it had to be frequently communicated that the panel speakers were to be children (no adults). • Meeting Face to face requires that the patient needs to be supported for physical accommodations. • Young people living with rare diseases may have urgent and unforeseen medical needs that prevent them from traveling, so it is helpful to increase the number of representatives. • During COVID-19, this type of meeting cannot be replicated as face-to-face meetings are not allowable for safety and health reasons.	https://bit.ly/38ZrpvW

We describe the overall lessons learned according to the PEQG criteria.

### Shared Purpose

The development of a shared purpose is fundamental to optimal outcomes of PPI. Researchers come with an intention which often needs to be shaped by mediators before it can become a purpose that is shared by the researcher and the participants in PPI.

All eleven activities describe some form of recruitment process prior to the PPI activity commencing to identify the most relevant candidates for the tasks. These processes start the process of establishing a shared purpose. The recruitment process was simpler for activities targeting members of an existing YPAG, entailing sharing the opportunity with the group/s, consenting for the activity to take place, and then supporting members throughout the activity. Most often, this entailed a considerable amount of correspondence and meetings between the researcher or research team and a facilitator who would then relay information to group members prior to the activity taking place. This iterative process allowed opportunity for facilitators and group members to shape a shared purpose before the activity started. If activities that were more ad hoc and/or required working with children and families with a particular expertise (i.e., the experience of living with a rare condition) this required additional steps in the process, such as production of flyers on how to get involved, expression of interest forms, terms of reference documents, or consent documentation that clearly explained the roles required. The terms of reference or consent forms detailed the aims of the activity, the remit and membership of the group, and other information (including payment and expenses, accountability, and confidentiality). The terms of reference or consent forms served to induct both children and parents into an activity. These forms were also used as a resource to fully inform participants about the activity and their role and to manage expectations regarding the activity throughout its course.

Lessons learned:

- Managing expectations of PPI for all parties requires a considerable amount of time prior to any activity taking place. Developing a shared purpose is fundamental for optimal outcomes.

### Respect and Accessibility

Regardless of the type of involvement chosen (i.e., focus group, consensus meeting, etc.), it was important that such activities were planned around the children and families’ schedules, either after work/school hours or during weekends, and not around the needs of research professionals’ availability (whenever possible). Voting polls circulated in advance of activities are helpful to identify convenient dates and times for children and parents to meet. Opportunities to attend via video teleconference/zoom should also be offered as alternatives to attending face to face, which became essential during the COVID-19 pandemic. Additional factors included making sure there was an allocated budget for meetings, reimbursement of travel, refreshments, and payment for children and parent contributions (although iCAN and some YPAGs do not pay their members but are reimbursed by the research team to pay the running costs of the networks). Those who did pay children and parents for their time found this quite complicated due to issues such as the requirement of having honorary contracts (for those aged 18 + and parents), tax and benefits issues. Paying children was particularly difficult so most YPAGs chose to offer gifts of appreciation in the form of ‘vouchers’ as a thank you for their contributions as opposed to cash or bank transfer (as not all children have bank accounts depending on their age). It is important to be open and transparent about payments or gifts of appreciation before involvement begins, and this can be achieved by having a payments policy in place that is agreed by all members.

Lesson learned:

- Being flexible around the timing of activities was seen to be the biggest factor in recruiting and retaining children and families throughout the activities.
- Realistic resources (including money, staff, time) should be allocated for PPI.

### Representativeness of Stakeholders

Diversity and representativeness of children and families is an issue not just for PPI, but for clinical research participation in general. It is essential that researchers consider who their target for participation is, which in turn will aid the decision on who needs to be involved. PPI facilitators are best placed to have a conversation with researchers and to organize the most suitable activity with the relevant stakeholders. Facilitators also support the selection of participants that meet the diversity profile agreed upon with the researcher. As the eleven case studies highlight, not all contributing children were members of a YPAG. When the case study facilitators opted for a different involvement model, they did this because of the condition being studied, which required specific input from children living with the disease to avoid tokenism. Under these circumstances, direct involvement of children living with a disease can have more impact on both the research design and on those who get involved.

Lessons learned:

- Organizing activities that involve those affected by a certain disease or condition requires more planning, time, and resources to ensure representativeness in terms of gender, disability, age, country diversity, and inclusion of children and families from disadvantaged socio-demographic backgrounds. This requires working in partnership with patient organisations and with clinicians working directly with patients and families affected by the disease.
- Involving both children and their parents in the activity provides a holistic view of the impact of the disease on the child and the family, but also requires additional planning to avoid parents dominating the conversations. A solution to this approach is to hold separate meetings for the children and adults, which requires not only more planning but also additional facilitators to manage and record the discussions.

### Roles and Responsibilities

The researcher is responsible for defining their requirements and identifying the resources needed to conduct the PPI activity. Ideally, the researcher can use feedback from facilitators and groups to make adaptions to activities before they take place. It is essential that the researcher sends timely feedback to participants involved in PPI activities about how their input has influenced the research or activity.

In some of the case study activities, researchers, or clinicians with expertise in the disease area or methodology were invited to join activity sessions to explain the study/project in detail and answer any questions that children and families had. This required many conversations between the facilitators and researchers before the activity took place so that everyone had a clear understanding of their roles during and after the sessions.

The facilitator’s role is to guide and support the researcher to make sure the planned activity is fit for their needs. Specific facilitator responsibilities may include activity design (i.e., selecting the best methodology for involvement), logistics (organizing meetings, facilitating discussions, etc.), and evaluation (reporting feedback to researchers, evaluating activities). More importantly, the facilitator’s role is to support children and families throughout the process so that their experience of involvement is a positive one and productive for all parties.

Lessons learned:

- A critical success factor is having skilled facilitators with experience working with children and families.
- Regardless of role, all participants in a PPI activity need to cooperate in the activity respectfully by complying to agreed role descriptions and terms of references. Having a clear memorandum between all parties, especially for long-term involvement activities is extremely helpful.

### Capacity and Capability for Engagement

Capacity and capability can be managed directly by skilled facilitators. Regardless of the activity, none of the case studies offered any formal (structured or accredited) training for children or families. That is not to say that formal training is not offered to children and families, but for the case studies a more flexible approach to learning was chosen and driven by individual needs and preferences. This included brief presentations and educational videos about a particular disease or research methodology during regular meetings, organizing topic-specific workshops (i.e., core outcome setting methods, etc.), and group discussions that generated a culture of learning and collaboration.

Lessons learned:

- Skilled, experienced facilitators offer a direct contact point of support to those who want to be involved in activities and to research teams with little experience of PPI.
- Flexible approaches to learning opportunities for children and families depends on individual needs and preferences.

### Transparency, Communications, and Documentation

Regardless of the PPI activity and type of involvement, it is clear from our experiences that tailoring communications to suit the needs of children (e.g., age and ability appropriate information) and families is essential. In some cases, the facilitators highlighted that recording in-depth notes for each PPI activity is important to capture what children and parents expressed. This required gaining permission for sessions to be recorded and transcribed for the purposes of publication (whether a report to the researcher or journal article), which is quite time-consuming. Another important consideration is to give participants feedback in a timely manner on how the study team acted upon their insights. Without this feedback, those who take part are left wondering about the value of their input and ultimately what impact it had on the activity.

Lessons learned:

- Tailored communication equipped children to get involved in activities and, more importantly, to stay involved and engaged throughout the process.

### Continuity and sustainability

Regardless of the length and type of activity, building meaningful relationships with children and families before, during, and after the activity is key. Children and families want to know that their time is valued, and their opinions are listened to and acted upon. Self-reflection, evaluation, and feedback mechanisms on the processes and value of the PPI activity are elements that need to be built into the activity from the very beginning. Sometimes these are an afterthought, resulting in missed opportunities to gather children, families, and researchers’ views of the strengths, weaknesses, and areas for improvement. At the very least, those who take part in activities should be provided with some written feedback about their contributions and thanked for their time and efforts. One of the biggest challenges for PPI facilitators is obtaining feedback in a timely manner. Realistic financial resources are also key to sustain PPI activities. Each PPI activity expands the experience of all participants and reduces the costs to future PPI activities.

Lessons learned:

- Self-reflection, evaluation and feedback mechanisms on the processes and the value of PPI need to be embedded into practice.
- Sustainability requires adequate financial resources.

## Discussion

There is an ongoing need to share examples of best practice PPI with children and families in the clinical research process to ensure that approaches are robust and meaningful to those who get involved. This paper reports on experiences of involving children and families at various stages of the clinical research process using a Patient Engagement Quality Guidance (PEQG) tool to guide reflections. The paper also links to a substantial corpus of projects that provide worked examples for PPI practitioners and people who commission PPI. The process of using the PEQG tool was an informative way of critiquing our experiences and practice of PPI with children and families. Systematic reflection on these experiences unearthed some important lessons and led to a comprehensive synthesis of lessons learned. These lessons contribute to the existing evidence base [6, 17, 18] by providing practical examples of how children, families, and researchers can work together, the difficulties encountered, and what is needed for meaningful PPI. The lessons learned about the process of involvement have shown that meaningful PPI requires support from skilled facilitators with experience of working with children and families and who can offer a direct point of contact to those who want to be involved in activities as well as research teams with little experience of PPI with children and families. Skilled facilitators can advise on the most suitable involvement approach and advise on planning child-appropriate activities, which saves a lot of time for researchers. This aligns to recently published findings [6] which highlighted the need for approaches adapted to each PPI activity. Furthermore, having these conversations early in the process helps to plan accordingly and review regularly how often children, and families will meet (i.e., around school/work and family commitments) and being flexible to the needs of those who want to be involved. This is particularly important for those affected by long-term chronic conditions who may not participate as frequently due to illness, medical emergencies, or caring responsibilities.

The lack of diversity within PPI is a well-recognized issue [19, 20] Therefore, it is essential during the planning process to consider the target group for a specific PPI activity. For some activities, the stability and expertise of a YPAG is useful. On the other hand, some of the case studies in this paper highlight that asking members of an existing YPAG is not always the best approach to gain children’s views, especially those living with certain chronic or rare conditions. As highlighted in previous literature [21, 22] this then requires looking at other approaches to involve those affected by the disease that is being studied, which in turn requires more planning, time, resources, and established links with key stakeholders (e.g., parent organisations, clinicians, charities, etc.). Another consideration is whether activities should include both parent’s and children’s views. If so, this requires careful planning and management of the activities to ensure that parents do not dominate the conversations. Regardless of the type or stage of involvement time and resources need to be invested to keep children and families motivated and engaged.

Skilled development training (i.e., in research methods, child rights/advocacy, communication skills, etc.) is one way to keep children and families motivated, especially for long-term projects/activities such as YPAG membership. However, similar to other published research findings [18, 23, 24] we found in some of the case studies that this level of training was viewed as unnecessary when children and families preferred informal conversational approaches to help them understand their roles. Thus, adopting a more flexible, informal induction into the activity with clear terms of reference, consent documentation and support from the PPI facilitator was felt to be sufficient.

Reimbursements are also a valuable and tangible demonstration of appreciation for children and families. No one should incur out of pocket expenses when taking part in PPI activities; at a minimum, travel and subsistence costs should be covered. This especially has implications for low-resource organisations with minimal budgets and impacts their ability to meaningfully involve children and families. One way to overcome this is to build in suitable budgets as part of grant applications to specifically support PPI activities, including budgeting for a skilled facilitator with experience working with children and families. Another way to keep children and families motivated is to provide feedback on the outcomes and impact of their input (both the impact on the study design and on the young people themselves). Van Schelven and colleagues (2020) also highlighted feedback as a motivational factor [17]. However, providing such feedback requires time to evaluate the activities, and incorporation of a clear tool or process for collecting and analyzing the feedback from participants, and researchers.

## Limitations

We note some limitations of this paper. The cases were gathered as a sample of convenience and not as a systematic survey. Each of the case studies had included an evaluation of children and families’ experiences of taking part in activities. However, the thematic reflections within the case studies were undertaken by the facilitators who had led the PPI activities. The timelines, effectiveness, impact, and the magnitude of the costs of PPI activities were not addressed in our analysis. Nevertheless, we believe that this paper provides useful insight into how to conduct PPI for industry and academic clinical researchers, and introduces a useful quality assessment for PPI with children and families.

## Conclusion

We no longer have to defend the view that involving children and families in the design and conduct of clinical research benefits both research and those who get involved. However, we must find ways to meaningfully involve children and families in these processes. Using the PEQF tool was helpful to self-reflect, capture, and share our learnings to guide future PPI projects with children and families. We suggest that the planning of future PPI projects will benefit from addressing the PEQF criterion to identify potential gaps prior to starting any PPI work with children and families. The lessons provided here provide a baseline for continuous improvement of the processes of PPI with children and families. High-quality PPI requires resources in time and money, skilled facilitators, and timely feedback from researchers.
